# Statement of Retraction: Dihydroartemisinin enhances the anti-tumor effect of photodynamic therapy by targeting PKM2-mediated glycolysis in esophageal cancer cell

**DOI:** 10.1080/14756366.2024.2385783

**Published:** 2024-08-09

We, the Editors and Publisher of *Journal of Enzyme Inhibition and Medicinal Chemistry*, have retracted the following article:

Mengru Jin, Luyao Shi, Dingyaun Zhang and Yanjing Li (2023) Dihydroartemisinin enhances the anti-tumour effect of photodynamic therapy by targeting PKM2-mediated glycolysis in oesophageal cancer cell, Journal of Enzyme Inhibition and Medicinal Chemistry, DOI: https://doi.org/10.1080/14756366.2023.2296695

Since publication, significant concerns have been raised regarding the Western blot images, ethical oversight of the study, and the authorship of the article.

When approached for an explanation, the authors provided responses to our queries and the original data but were unable to address all of the concerns raised.

As verifying the validity of published work is core to the integrity of the scholarly record, we are therefore retracting the article. The corresponding author listed in this publication has been informed.

We have been informed in our decision-making by our Editorial Policies and COPE guidelines.

The retracted article will remain online to maintain the scholarly record, but it will be digitally watermarked on each page as ‘Retracted’.

